# Research on the Intelligent Display of Cultural Relics in Smart Museums Based on Intelligently Optimized Digital Images

**DOI:** 10.1155/2021/7077556

**Published:** 2021-12-15

**Authors:** Yutian Sha, Shaohan Zhang, Tianxin Feng, Ting Yang

**Affiliations:** Tongji University, Shanghai 200092, China

## Abstract

The smart museum is a new platform for the appreciation and display of cultural relics across time and space. In the era of 3D scanning technology, computer technology, and network technology, it is necessary to deeply study the smarter and more perfect forms of smart museums. This article, through the analysis of the characteristics of the display level of the smart museum, tries to create a new humanized and intelligent display of cultural relics.

## 1. Introduction

China's cultural relics are quite colorful, but the museum resources enjoyed by the Chinese people are limited. At the same time, the distribution of these museums is uneven. In today's information age, if only traditional museums cannot meet people's needs, the development of intelligent museums is imperative for cultural dissemination, preservation of historical materials, sharing of cultural relics, and academic research [1–3]. For the preservation of data, the sharing of cultural relics and academic research, etc., it is imperative to develop an intelligent museum. The Wisdom Museum is a brand-new platform for displaying cultural relics and smart technology. It stimulates customers' love through mutual communication with art so that customers can understand the true meaning of cultural relics. In addition, the smart museum can also display exhibits of their own choosing according to the different hobbies of the audience. Using this interactive form, Nengou allows the function of education to play its greatest role and at the same time enhances the spirit of self-learning of visitors. The characteristic of the intelligent exhibition hall is that it can provide diversity and multifacetedness, and at the same time, it shows the characteristics of dynamics and different angles when researching and designing. With the rapid development of intelligence, information about cultural relics and exhibits in museums is becoming more and more important to the museum's modules [4–6]. Intelligent optimization algorithms are developed based on virtual reality, which “enhance” the real world by superimposing computer-generated virtual objects, scenes, or system prompt information on the actual scenes. The intelligent optimization algorithm can not only introduce the virtual object into the actual environment, but also dynamically give the position and posture of the virtual object to maintain the consistency of the virtual object and the actual scene. Form the habit of observing the intelligent optimization algorithm environment roaming with more personal eyes, this way of interaction looks more natural. On the other hand, since the intelligent optimization algorithm maintains the actual scene, the output result is more realistic. The intelligent optimization algorithm can use the camera to capture the real situation to obtain the video stream. The tracking method is used to process each frame in the video stream, the actual coordinates and state of the camera are calculated using geometric calculation methods, and the coordinates and states of the virtual objects registered in the actual background (also known as three-dimensional registration) form a virtual scene. Using the technology of video combination, the virtual scene and the real background video stream are combined, and the combined result (video stream) is transmitted to the display in time.

The main work of the museum is the display of exhibits and the technology and means of educational functions. With the continuous innovation of technology, the smart museum has many shortcomings in the display mode. This article analyzes the characteristics of smart museums and attempts to further study the mode of smart museums' intelligent display.

## 2. Overview of the Development of Smart Museums

**Figure 1:**
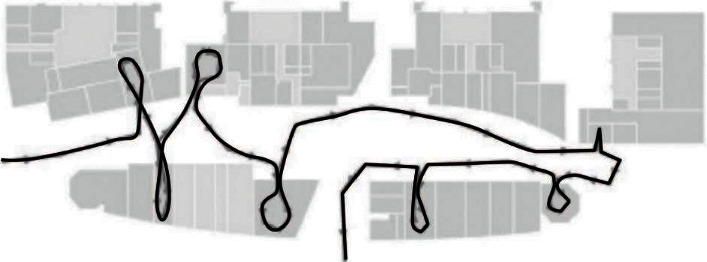
User's roadmap.

With the continuous development of analysis technology and intelligent optimization technology of large museums, the new intelligent display mode of intelligent museum cultural relics has gradually been used in many fields. From the research status at home and abroad, the interface designed for the new intelligent display of intelligent museum cultural relics can be analyzed. The transformation shows the changing trend of heterogeneity and the presentation form of two-dimensional modularization. According to the optimization of the original intelligent display mode, it can be concluded that almost all new intelligent display modes are search results for homogeneity and cannot accurately describe the real topology. The trajectory line is displayed on the map, and the trajectory line is mainly connected with each other by real-time points, and there is a floor selector on the left side of the map [7–9]. Below the map, there is a user list, user mac address and number, etc.  Figure 1 shows the user's route map.

All the visitors on the map are well displayed. The position of the personnel is marked with a red dot. You can select floors on the left side of the map. In addition, you can count the number of people in the museum during a certain city time period and classify the visitors according to the time spent in the museum. (e.g., the positions of people staying at the booth for more than 20 minutes). At the same time, it supports more than 10,000 client terminals and uses location services to effectively call the location information of each viewer. Analyze the passenger flow of the museum, adjust the location engine to the background update frequency of the museum center, and record the terminal location information. Configure all terminals on the floors, separate floors to display floor trajectories, and add up customer trajectories in a user-defined time period.

There are various types of cultural relics related to the Great Wisdom Museum with a new mode of intelligent display of cultural relics in the Smart Museum, but the amount of cultural relics in the Smart Museum is a large-capacity museum. If you need to complete a large-capacity cultural relic display, you need to divide the cultural relics of the smart museum. In the exhibition space of the museum, it is better to divide the corresponding subcategory under the cultural relics catalog of the wisdom museum [10–12]. At the same time, display the subcategory to the corresponding management location. In addition, to display illustrations and other media museums, it is necessary to mark the illustrations at the entrance and exit of the museum so that they can be restored during output.

In this article, regarding the use of intelligent optimization technology to display cultural relics, intelligent optimization technology is characterized by the work of cutting out the cultural relics from the back from the end of the cultural relics. The traditional display method of cultural relics is to display and extract in the order of the catalog, copy the contents of the subdirectories, and display the copied content in the order of pasting the copied content to the new empty cultural relics. Save to the museum library on the homepage. However, if large cultural relics are displayed in sequential display, if the capacity of a single cultural relic reaches GB, its display efficiency will be reduced, which may cause the collapse of the system museum. The reason is that the entire cultural relics are opened in the museum, and operations such as copying, pasting, saving, and uploading consume a lot of museum space. In the document, the cultural relics are cut from the back, and the cutting of the cultural relics will gradually reduce the display space occupied by the cultural relics. Therefore, if you do display operations, the display speed will become faster and faster. However, if they are displayed in order, the content of the cultural relics will move every time they are cut, and the form of the cultural relics will be chaotic, and a satisfactory display effect may not be obtained.

### 2.1. Advantages and Limitations of Smart Museum Display

So far, when using this kind of technology to promote the virtual reality of the intelligent display of cultural relics in the smart museum design, there are many limitations, such as important technology and light aesthetics. Particularly, in many cases, we pay special attention to the development and research of intelligent optimization algorithms, ignoring the actual needs of intelligent museum design for the intelligent display of cultural relics. There is a big gap with actual needs. In addition, there are also problems in dealing with the environmental art of the intelligent display of cultural relics designed by the smart museum. All smart museums are not isolated elements. The complete smart museum design and intelligent display of cultural relics contain specific smart museum objects and the surrounding environment (e.g., mountains, rivers, confluences, and trees). Therefore, in order to construct a perfect model, not only the hardware equipment, but also the intelligent optimization algorithms used in specific applications also provide high requirements. In order to reproduce the environmental aesthetics described in it more realistically, the picture quality is very good and smooth. This is a big challenge for current intelligent optimization algorithms. Judging from the status quo of the real-scene virtual reality display of the intelligent display of cultural relics designed by the smart museum, the real-scene virtual reality of many models only focuses on the main body of the smart museum, ignoring the adaptation to the surrounding environment. Therefore, the virtual reality effects of many real scenes do not conform to the original intention of the intelligent museum to design cultural relics.

For smart museums, remote protection is a passive compromise. Virtual reality of the channel is not protection itself; it is a technical means to avoid disassembly simulation. The core and goal of the actual virtual reality strategy is how to maximize the protection of the smart museum. The mobile smart museum is not a tool to solve the contradiction between planning and construction, but a device that can be moved as needed. The different starting point and perspective determine the choice of value and the future direction of development. There is a fundamental difference between the virtual reality of the actual scene of the smart museum and the general smart museum.

Compared with traditional museums, smart museums have the same basic functions, displaying and displaying cultural relics, but the realization methods are quite different. Traditional museums require visitors to go directly to the museum to see the cultural relics. In addition, it is impossible to fully appreciate the content of the exhibits. The analysis factors corresponding to the subject to be analyzed are used as elements, and the above elements are used to construct an analysis factor set. Based on the intelligent optimization algorithm, the intelligent museum cultural relics intelligent display system design method obtains analysis standards according to the needs and characteristics of the analysis object and constructs the analysis factor set of the intelligent museum cultural relics intelligent display.

Based on the intelligent optimization algorithm, the intelligent museum cultural relics intelligent display system design method divides the analysis factor set, and the first-level analysis factor set is obtained [13–15]:

(1) $$\begin{array}{c} U=\left\{U_{1},U_{2},\cdots ,U_{m}\right\}. \end{array}$$

The first-level analysis factor set of the intelligent display of cultural relics in the smart museum is further divided, and the second-level analysis factor set is obtained:

(2) $$\begin{array}{c} \left\{\begin{array}{c} U_{1}=\left\{U_{11},U_{12},\cdots ,U_{1n}\right\}\\ U_{2}=\left\{U_{21},U_{22},\cdots ,U_{2n}\right\}\\ \vdots \\ U_{m}=\left\{U_{m1},U_{m2},\cdots ,U_{mn}\right\} \end{array}\right.. \end{array}$$

Combine the characteristics of the intelligent display factors of cultural relics in each smart museum, analyze the actual situation of the intelligent display of cultural relics in smart museums, and divide the quality of multimedia professionals into four levels: excellent, good, medium, and poor:

(1) Comparing the indicators existing in the same layer, construct the judgment matrix *A*, the expression of which is as follows:
  (3) $$\begin{array}{c} A=\left[\begin{array}{cccc} a_{11} & a_{12} & \cdots & a_{1n}\\ a_{21} & a_{22} & \cdots & a_{2n}\\ \vdots & \vdots & \; & \vdots \\ a_{n1} & a_{n2} & \cdots & a_{nn} \end{array}\right]. \end{array}$$
Normalize the judgment matrix by column:
  (4) $$\begin{array}{c} b_{ij}=\frac{a_{nn}}{\sum_{i=1}^{n}a_{nn}}. \end{array}$$
Normalize the judgment matrix by row:
  (5) $$\begin{array}{c} v_{ij}=\sum_{h=1}^{n}b_{ij}. \end{array}$$
Suppose *w*_*i*_ is the weight corresponding to the intelligent display index of cultural relics in the smart museum, and its calculation formula is as follows:
  (6) $$\begin{array}{c} w_{i}=b_{ij}\cdot \frac{v_{i}}{\sum_{i=1}^{n}v_{i}}. \end{array}$$
The weight vector matrix *W* is constructed according to the calculated index weights of the smart museum cultural relics intelligent display.
(2) Judge the consistency of the decision matrix with a high step number through the consistency check.

According to the collected museum analysis, the following is constructed:

(7) $$\begin{array}{c} U_{mn}=\left[\begin{array}{c} U_{11},U_{12},U_{13},U_{14},U_{15}\\ U_{21},U_{22},U_{23},U_{24},U_{25}\\ \vdots \\ U_{m1},U_{m2},U_{m3},U_{m4},U_{m5} \end{array}\right]W. \end{array}$$

According to the calculated degree of membership, the intelligent museum cultural relic display model *F* is constructed, and its expression is as follows:

(8) $$\begin{array}{c} F=\frac{w_{i}\times U_{mn}}{U_{j}}\times \delta , \end{array}$$

where *δ* is the fuzzy operator.

The intelligent optimization algorithm-based smart museum cultural relics intelligent display system design method uses the intelligent optimization algorithm to solve the constructed smart museum cultural relics intelligent display model and realizes the fuzzy comprehensive analysis of the quality of multimedia professionals. Calculate the Euclidean distance that exists between two weight vectors. According to the calculation results, the *T* vector is selected as the adjacent part of the weight vector.

Suppose *B*(*i*)={*i*_1_, *i*_2_, ⋯, *r*_*T*_}, *i*=1,2, ⋯, *N*; *λ*^*i*_1_^, *λ*^*i*_2_^, ⋯, *λ*^*i*_*r*_^, plus describing the *T* weight vectors with the closest distance around the uniformly distributed weight vector *λ*^*i*^.

Initialize the population *x*^1^, *x*^2^, ⋯, *x*^*i*^; set *F*(*x*^*i*^)=1/*λ*^*i*_*r*_^*F* · *B*(*i*).

Divide the population *P* to obtain 3 subpopulations IA, IB, and IC so that there are *ξ*_1_ individuals in the subpopulation IA, *ξ*_2_ individuals in the subpopulation IB, and *ξ*_3_ individuals in the subpopulation IC. Set *ξ*_1_, *ξ*_2_, and *ξ*_3_ meet in the initial stage:

(9) $$\begin{array}{c} \xi_{1}=\xi_{2}=\xi_{3}=\frac{N}{B}. \end{array}$$

The dynamic collaborative differential evolution is carried out through the dynamic subgroup method, so as to show the design method of the intelligent museum cultural relic intelligent system based on the intelligent optimization algorithm. The specific process is as follows:

(1) Obtain a new individual *y*^*i*^ of the offspring.
(2) Update the reference point.
(3) Evolution success rate is
  (10) $$\begin{array}{c} \tau_{1}=\frac{\kappa_{1}/\xi_{1}}{\kappa_{1}/\xi_{1}+\kappa_{2}/\xi_{2}+\kappa_{3}/\xi_{3}},\\ \tau_{2}=\frac{\kappa_{2}/\xi_{2}}{\kappa_{1}/\xi_{1}+\kappa_{2}/\xi_{2}+\kappa_{3}/\xi_{3}},\\ \tau_{3}=\frac{\kappa_{3}/\xi_{3}}{\kappa_{1}/\xi_{1}+\kappa_{2}/\xi_{2}+\kappa_{3}/\xi_{3}}. \end{array}$$

Here, *κ* is the evolution strategy corresponding to different individuals.

In addition, the functional distinction is insufficient. In order to consider the security of cultural relics museum information and text information, an excellent hierarchical management module is set up according to the reading purpose, and visitors are classified into scholars and general readers. Different users who log into the museum provide different levels of authority. The “Notice of the State Council on Strengthening the Display of Cultural Heritage” proposes for the first time that it is necessary to “fully understand the importance of displaying cultural heritage from the perspective of maintaining national cultural security.” Although the related information of the collection does not belong to the category of museum collections in the traditional sense, in the future work of the museum, the collection itself and related information have in fact occupied the same important position. By applying the intelligent optimization algorithm to the new mode of intelligent display of museum cultural relics, it can solve the current inability to obtain interesting information for users and improve the efficiency and accuracy of the new mode of museum cultural relics. At the same time, the intelligent optimization algorithm is used in the new mode of the museum's cultural relics intelligent display to achieve the real connection between big data information.

### 2.2. Application of Intelligent Optimization in the New Mode of Intelligent Display of Cultural Relics in Smart Museums

In addition to meeting personal functional requirements, a reasonable smart museum design should also maintain good coordination with the surrounding environment. Now is the era of intelligence, and using intelligent optimization algorithms in the actual three-dimensional display of cultural relics designed by smart museums can give better play to its technical advantages. It also represents the trend and status of the performance and significance level of the intelligent display of cultural relics designed by the smart museum (especially the stable development status of the introduction of virtual technology); the model real-life 3D TV adopts intelligent optimization algorithms to respond to the development of the current era. The focus of the modern industry is the virtual reality research situation of the virtual three-dimensional smart museum. The Virtual Wisdom Museum has mathematical modeling, geometric warehouse, texture and rendering, etc. At the same time, every constructing mathematical modeling and geometric programming in the smart museum has great challenges. The virtual wisdom museum in the large-scale wisdom museum has a great use in traditional parades and actual real estate demonstrations. Currently, researchers at home and abroad have carried out many research activities based on imitating the virtual wisdom museum. The research on OpenGL's assistance, modeling, and simulation motion composition in three-dimensional virtual explained the actual motion level and data and completed the modeling and simulation of the smart museum under virtual conditions. Because the virtual reality smart museum design multimedia design and simulation system has limitations, this article proposes to apply the smart optimization algorithm to the smart museum design multimedia design and simulation system. Ensure that the advantages of the virtual reality smart museum simulation system are not destroyed, help designers deal with these two difficulties, improve design efficiency, and give a fair evaluation of the smart museum design plan.

**Figure 2:**
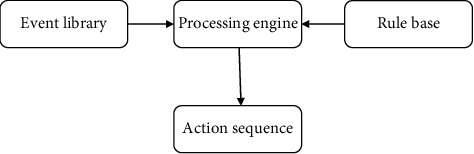
Intelligent display of the overall framework of the new model.

In this article, we suggest using intelligent optimization algorithms to construct an intelligent display combined with the characteristics of the subject, and the current intelligent display cannot provide users with interesting information, thereby improving the efficiency of the new model. Use the host to make the intelligent display have the characteristics of intelligent retrieval as the theme of the intelligent museum's cultural relics. The entity knowledge model can provide search results that are of interest to users. The project uses a new mode of intelligent display combined with intelligent optimization algorithms to realize the design of intelligent display. The overall framework of the intelligent display of the new model is shown in Figure 2.

The event library will display the collected museums as basic events waiting for the engine to process. The developer's configuration rules should be stored in the rules warehouse. The processing engine reads the basic events and business rules through the event interface and the rule interface, respectively, and generates the event according to the processing algorithm. Whether the event meets the rule. The processing method is that if the event that occurs meets the rule, the action is triggered.

The intelligent display workflow based on intelligent optimization algorithm optimization is as follows:

1. According to the user's search conditions, the text documents related to the keywords input by the user are obtained from the engine museum library as an initialization group and displayed to the user.
2. The user selects the highest item that is qualified or not according to the text file he needs.
3. The engine calculates the text document with a higher numerical evaluation of the objective function as the preferred category and returns the text document selected by the user to the user based on the iterative step calculation introduced by the basic flow of the above intelligent optimization algorithm.
4. If the user finds the text document required by the text document returned from the engine, the new mode is stopped. Otherwise, we will proceed to step (2).

**Figure 3:**
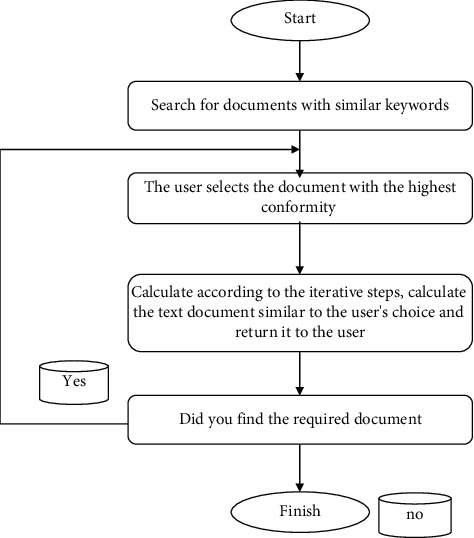
Intelligent display of the new model process.

The intelligent display optimized by the intelligent optimization algorithm can enable users to participate in the new mode process so that the results of the new mode are closest to the needs of the users, and the accuracy and satisfaction of the results of the new mode can be guaranteed. The flow chart of this action is shown in Figure 3.

For most intelligent tutoring systems, the ITS of smart museum cultural relics consists of three main parts: (i) knowledge base, (ii) counselor, and (iii) a new mode of intelligent display of museum cultural relics. One of the main differences from other ITS is that in this case, there will be no direct evaluation of the museum with questions or problems. According to the new model display results and exploratory behavior, when indirectly evaluating the museum, the designer must evaluate the museum's cognitive status and decide the best display activity. With this information, designers must evaluate the cognitive state of the museum and decide on the best exhibition activities. Taking into account the inherent uncertainty of this task, a new mode of intelligent display of cultural relics has been developed for the smart museum. The attributes of entities and the relationships between them represent the domain. Next, I will give a brief introduction to PRM and then discuss their application in the new mode of intelligent display of museum cultural relics.

The new mode of intelligent display of cultural relics in the smart museum the basic entity in the new mode of intelligent display is the object or domain entity.

According to other variables in the new intelligent display mode, different Bayesian networks can be generated from the bones. For example, in the new mode of intelligent display of museum cultural relics described below, a general framework of experiments is defined, from which specific examples of each experiment can be generated. This provides greater flexibility and versatility for intelligent display of new models, thereby facilitating knowledge acquisition. Since only a part of the new model is presented intelligently in each specific situation, this also makes reasoning more effective. This new mode of intelligent display specifies the main categories of objects and their dependencies. For example, the class knowledge item represents the museum's knowledge of specific concepts in the experiment. This knowledge is related to the results of the experiment and the behavior of the museum and will affect the museum's knowledge at a higher level (subtopics and topics). PRM uses the same as Bayesian networks. The basic principle specifies the probability distribution of bones. It is assumed that each random variable in the PRM, in this case, the attribute x ∈ a of a single object *x*, is only directly affected by a few other variables.

### 2.3. Design of the Cultural Relics Display of the Wisdom Museum

#### 2.3.1. System Design Requirements

This system is an independent system. The main purpose is to combine the wisdom museum and geography to make the main model of immovable cultural relics so that visitors can open the software to see the cultural relics.

Based on the user's perspective analysis, the system is designed for the user of personnel without the professional background of cultural relics, because the user can easily understand the cultural relics and materials of interest in the humanized and simplified design in the appearance and use method of the interactive interface between man and machine.

Based on the performance analysis of the system, the system is required to have operability, standardization, scalability, and compatibility.

#### 2.3.2. System Architecture Design

Wisdom museum objects have material and spiritual values. There are two reasons for the continuation of smart museum objects. The lifespan of the material itself is inconsistent with the lifespan of the society. The lifespan of smart museum objects is different, and the lifespans of the components of smart museum objects are also different. In the past wooden wisdom museum, the parts that need to be repaired and replaced. Among the above, the displacement requirements of smart museum objects are adjusted according to the land use, and there are many standards related to the construction of the city. The three-dimensional visual significance of the real scene of the common smart museum is to continue to promote its material value and ultimately pay more attention to the calculation of transfer and construction costs. Wisdom museum objects are the continuation of material life, and the meaning of their existence is to focus on the material. There is still a current situation that the use time limit of many smart museum objects has exceeded their regular time limit. If the purpose of its protection is brought into play, it must first be supported and resonated by people. If the value of the smart museum object decreases or even does not exist, personal emotions, deeds, historical records, and other factors have produced the desire to keep this smart museum. This is the meaning of virtual reality protection of real scenes. In addition to the meaning of social existence and the value of the material itself, the value of life and the issue of separation, in the context of historical and social cultural value, the strategic significance of virtual reality of smart museum objects is to maximize the protection and inheritance of smart museum objects.

In order to facilitate the comprehensive utilization of information, we propose a smart museum structure based on the B/S three-tier structure, which combines multiple elements such as strength and function. From top to bottom are the layer, control layer, and museum library layer. The main purpose of the view layer is to enable the system to interact with the user. The user sends a service request corresponding to each layer and forwards the request to the next layer. The view layer indicates the result to the user according to the return request from the next layer. The control layer realizes system functions and can realize logical control. This layer is mainly composed of mutually independent parts, which, respectively, activate the display map module and the meaning inference module. The semantics module implements reasoning according to the corresponding rules in the regional knowledge base and commands the retrieval of information about the semantics of cultural relics in the nonspatial museum library. The main purpose of the map module call is to send a request to the space museum library and return a map of cultural relics. The library layer of the museum mainly contains the needs of the system, the museum of cultural relic meaning information, and the museum of geographic information, which belong to the basis of the system [3].

## 3. Experiment and Result Analysis

Intelligent optimization indoor positioning is the same as wifi positioning, and the RF field strength is calculated in the distance attenuation model. Intelligent optimization is based on the topology of the Bluetooth base station, and the signal strength is expressed by the RSSI value. People in the museum can use the change of the RSSI value to judge the distance between the intelligent terminal and the intelligent optimization device. This value is a rough information; even if it is nearby, not available, or within a long distance, the rice can be reached accurately. If the intelligent optimized base station is arranged in space every 30m, the position accuracy can be maintained at the decimeter level, which fully meets the daily location requirements of the museum.

In the intelligent optimization algorithm, the new mode is (*g*, *n*), among which *n*=*p* × *q*; the mode is

(11) $$\begin{array}{c} \lambda =1cm\left(\left(p-1\right),\left(q-1\right)\right). \end{array}$$

**Figure 4:**
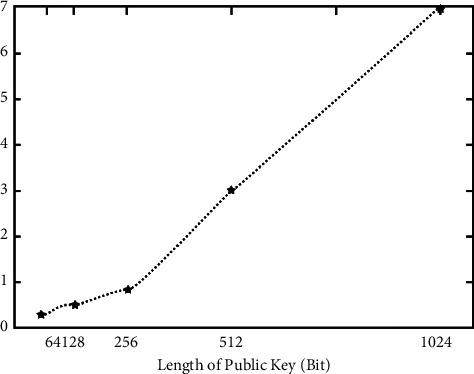
Cultural relics show the generation time of the new model.

In the experiment, 64, 128, 256, 512, and 1024 bits are generated to show the new mode and the corresponding *g* and *λ*, respectively. Because of the gradual increase of these lengths, the amount of calculation in the generation will increase one by one, and the corresponding growth rate will also increase, as shown in Figure 4.

**Figure 5:**
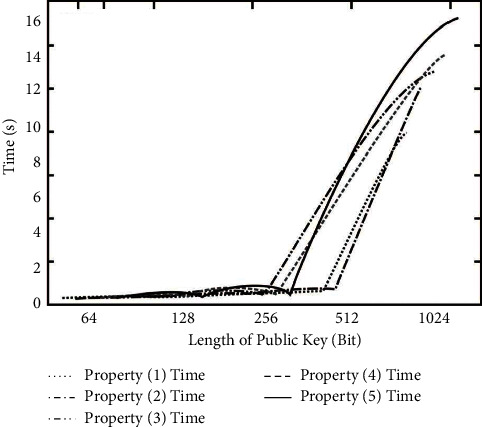
Display time of the new mode of cultural relics.

Figure 5 shows the evaluation results after comparing additive homomorphisms and multiplicative homomorphisms. It can be seen that the intersection of additive homomorphisms is more time-consuming.

The intelligent display of cultural relics in the smart museum can make the audience feel a more humane and comfortable real experience. The positioning requirements in the hall are the two aspects of exhibit positioning and route planning. After the intelligent optimization equipment obtains the real-time position of the audience, it uses the new model to show the audience the storage of cultural relics in each exhibition hall. From the analysis of the research results in this article, it can be concluded that the use of the new model technology for the intelligent display of museum cultural relics can solve the current inability to obtain interesting information for users, improve the efficiency and accuracy of the new model of museum cultural relics, and solve the problem of one network. The simple new mode cannot obtain the problem of information needed, but because the data synchronization technology needs to import data information after each restart on the client side, the loading time on the user side is longer, and further optimization is required.

## 4. Conclusions

The intelligent display of cultural relics designed by smart museum uses modern technology for protection and simulation display. The intelligent display of cultural relics designed by smart museum not only constructs the correct three-dimensional model in its report materials, but also pays attention to the required artistic beauty. Therefore, we have seen the success of the intelligent display of cultural relics in smart museums with real-life three-dimensional visualization. More effects such as “pictures are stiff” and “lack of connotation” are displayed, making the multimedia unreal engine technology not fully utilized, no matter it is a smart museum. The actual three-dimensional visual art effect of the intelligent display of cultural relics in design, or the aesthetic perspective, is very different from the intelligent display of cultural relics in the smart museum. With the rapid economic development, people's living standards are improving day by day, and their spiritual needs continue to increase. Museums are the main platform for the spread of knowledge, cultural customs, etc., and are deeply loved by tourists. It is just that the current museum form in our country is in the traditional mode, and the main job is to preserve and research cultural relics, which cannot meet the various needs of tourists. For this phenomenon, this article puts forward the form of humanized museum cultural relics display to improve the effect of tourists' appreciation of museums.

## Data Availability

Data sharing is not applicable to this article as no datasets were generated or analyzed during the current study.
